# Important Discovery by the Microscope

**Published:** 1888-10

**Authors:** 


THE FOLLOWING SCIENTIFIC (?) ITEM goes to prove a statement made by the late Professor Julius Miner, "A man can see whatever he is looking for with a microscope": A well-known New York physician has just published the sort of discovery which Lord Lytton would have made a novel out of. An aged Polish count, formerly professor of languages and a famous oriental scholar, died in the hospital, and Dr. Rockwood had occasion, in conjunction with other experts, to make a microscopical examination of a certain part of the cerebrum. They noticed a peculiar set of markings, which took the form of Egyptian and Chinese hieroglyphics. These were amplified to a magnitude of 3,000 diameters, and the results shown to another oriental scholar, who declared them to be true characters in the Ethiopic, Syric and Egyptian languages. Dr. Rockwood suggests that his discovery will lead to extracting from the dead their literary achievements as well as their "suppressed opinions."—*Hardwicke's Science-Gossip.*

